# Psychotic symptom burden and serum uric acid as correlates in a limited-adjustment analysis of current suicidal ideation in schizophrenia: a cross-sectional study

**DOI:** 10.3389/fpsyt.2026.1921230

**Published:** 2026-10-22

**Authors:** Huimin Chen, Jian Lv, Yu Xu, Jinchang Tian, Xiang Liu

**Affiliations:** 1 Department of Geriatric Psychiatry, Shanghai Jing’an District Mental Health Center, Shanghai, China; 2 Department of General Psychiatry, Second People’s Hospital of Jiangning District, Nanjing, Jiangsu, China

**Keywords:** beck scale for suicide ideation, biomarker, cross-sectional study, positive and negative syndrome scale, psychotic symptoms, schizophrenia, serum uric acid, suicidal ideation

## Abstract

**Background:**

Suicidal ideation is common in schizophrenia, but simple approaches combining symptom severity with routine biochemical measures remain limited. Serum uric acid, the end product of purine metabolism, is a routinely available peripheral measure linked to antioxidant activity, oxidative-inflammatory processes, and metabolic pathways, providing a rationale for its exploratory evaluation alongside psychotic symptom burden. We examined limited-adjustment associations of psychotic symptom burden and serum uric acid with current suicidal ideation in patients with schizophrenia and exploratorily evaluated the apparent discrimination of a combined model.

**Methods:**

This cross-sectional study included 134 patients with schizophrenia treated at Shanghai Jing’an District Mental Health Center from May 2021 to October 2023. Current suicidal ideation was defined as any nonzero response on item 4 or 5 of the Chinese Beck Scale for Suicide Ideation/Beck Scale for Suicide Ideation-Chinese Version (BSS/BSI-CV) during the preceding week. Psychotic symptoms were assessed using the Positive and Negative Syndrome Scale (PANSS). Fasting blood samples were used to measure routine biochemical indicators. Between-group comparisons, prespecified limited-adjustment logistic regression, and auxiliary ROC analyses were performed. Data on body mass index, renal function, detailed medication exposure, urate-related treatment, and diet were unavailable.

**Results:**

Current suicidal ideation was identified in 23 patients (17.2%). Patients with suicidal ideation had higher PANSS total, positive, and general psychopathology scores and higher serum uric acid levels. In the model adjusted only for age and sex, PANSS total score (odds ratio [OR] per 10-point increase = 2.05, 95% confidence interval [CI]: 1.27–3.31; *p* = 0.003) and serum uric acid (OR per 100-µmol/L increase = 2.46, 95% CI: 1.16–5.25, *p* = 0.019) remained associated with current suicidal ideation. Among the three ROC analyses, the combined model yielded the highest AUC (0.768, 95% CI: 0.657–0.865).

**Conclusion:**

Greater psychotic symptom burden and higher serum uric acid were associated with current suicidal ideation in schizophrenia. Serum uric acid should be interpreted as an exploratory biochemical correlate, not a mechanistic risk factor or validated biomarker. The model cannot be used for clinical prediction without further validation.

## 1 Introduction

Schizophrenia is a severe psychiatric disorder characterized by disturbances in perception, thought, emotion, cognition, and behavior. According to the World Health Organization, schizophrenia affects approximately 23 million people worldwide and is associated with substantial disability, stigma, human-rights violations, and premature mortality (1). Contemporary reviews highlight the biological and clinical heterogeneity of schizophrenia, including abnormalities in neurochemical signaling, synaptic development, neural circuits, cognition, and functional outcomes (2–4). Mortality in schizophrenia is markedly elevated, and suicide-related outcomes remain among the most serious contributors to premature death and disease burden (5, 6).

Suicidal ideation is clinically important because it may precede suicide attempts, death by suicide, and recurrent crisis presentations. A recent systematic review and meta-analysis of Chinese patients with schizophrenia reported pooled estimates of lifetime and point prevalence of suicidal ideation of 19.22% and 18.06%, respectively, among high-quality studies (7). Worldwide epidemiological evidence also indicates that suicidal ideation and suicide plans are common in schizophrenia (8), and recent large-scale Chinese studies continue to show that current suicidal ideation is a frequent problem in chronic schizophrenia (9). These data support the need for routine, structured screening of suicidal ideation in both inpatient and outpatient psychiatric settings.

Suicide-related vulnerability in schizophrenia is multidimensional. Recent studies have linked suicidal ideation with Positive and Negative Syndrome Scale (PANSS) total score, positive symptoms, general psychopathology, insomnia, depression, aggression, neurocognitive function, empathy, attention, and emotion-related impulsivity (10–19). The PANSS is widely used in clinical practice and research, and its total and subscale scores may capture symptom dimensions related to acute distress, perceived threat, agitation, impaired coping, and loss of behavioral control. However, symptom scales alone may not fully reflect the biological milieu accompanying suicide-related vulnerability.

Peripheral biomarkers related to purine metabolism, oxidative stress, inflammation, lipid metabolism, endocrine function, and metabolic dysregulation have increasingly been investigated in schizophrenia and suicide-related research, including lipid-related measures (20). Uric acid, the final product of purine metabolism, is a major endogenous antioxidant but may also reflect oxidative-inflammatory activation or metabolic burden in specific clinical contexts. Recent studies have suggested that serum uric acid is associated with systemic inflammation, symptom dimensions, and cognitive function in schizophrenia (21–23). In parallel, oxidative-stress parameters, cytokine activity, inflammatory biomarkers, lipid-related markers, thyroid hormones, vitamin D, and broader metabolic profiles have been examined as potential correlates of psychiatric symptoms and suicide-related phenotypes (24–37).

Despite these advances, clinically feasible approaches that integrate psychotic symptom burden with routine biochemical indicators remain limited. Recent reviews and reporting guidelines emphasize that multivariable models in psychiatry should be interpretable, transparently reported, and cautiously interpreted when validation is unavailable (38, 39). Medication-related metabolic and biochemical effects also complicate biomarker interpretation in psychiatric populations (40). This issue is particularly important for serum uric acid, which is strongly influenced by renal function, body mass index, diet, alcohol use, urate-lowering or diuretic treatment, and antipsychotic-related metabolic effects. When these determinants are unavailable, uric-acid findings should be framed as limited-adjustment associations rather than evidence of a biological mechanism. Recent work using passive sensing and multidomain clinical–biological approaches highlights the promise of integrated risk stratification while underscoring the need for optimism correction, calibration assessment, decision-curve analysis, and external validation before any model is considered clinically useful (41, 42).

Beyond schizophrenia, serum uric acid has been examined across affective disorders and suicide-related phenotypes, although the direction of association is not uniform. A 2023 meta-analysis in bipolar disorder reported higher serum uric acid levels than those in patients with depression or schizophrenia and in healthy controls, with higher levels during manic than depressive episodes (43). In major depressive disorder, a meta-analysis found lower serum uric acid on average but no evidence of a causal association in Mendelian randomization analyses (44), while a retrospective study of adolescents and young adults with major depressive disorder reported lower uric acid among those with recent suicide attempts (45). These cross-diagnostic findings suggest that uric acid may reflect disorder-, state-, sex-, metabolic-, or treatment-dependent processes rather than a diagnosis-specific biomarker. Biologically, uric acid also has context-dependent redox and inflammatory properties. Although it is an important circulating antioxidant, experimental studies indicate that soluble uric acid can promote reactive oxygen species generation and activate TLR4/NF-κB/NLRP3 inflammasome signaling, with downstream interleukin-1β release (46). This provides a plausible inflammatory rationale for studying uric acid in psychiatry, but such mechanisms cannot be inferred directly from serum uric acid concentrations in the present cross-sectional study. Therefore, the present study examined limited-adjustment clinical and biochemical correlates of current suicidal ideation in patients with schizophrenia and included ROC analysis only as an auxiliary exploratory description of the apparent performance of an age- and sex-adjusted logistic model combining PANSS total score and serum uric acid.

## 2 Methods

### 2.1 Participants

This observational study included 134 patients with schizophrenia who were treated in the inpatient and outpatient departments of Shanghai Jing’an District Mental Health Center between May 2021 and October 2023. The diagnosis of schizophrenia was established according to the criteria of the tenth edition of the International Classification of Diseases (ICD-10). According to the prespecified definition of current suicidal ideation during the preceding week based on Beck Scale for Suicide Ideation/Beck Scale for Suicide Ideation-Chinese Version (BSS/BSI-CV) items 4/5, patients were classified into a suicidal-ideation group and a nonsuicidal-ideation group. The inclusion criteria were as follows: (1) meeting the ICD-10 diagnostic criteria for schizophrenia; (2) being 18–65 years of age; and (3) providing written informed consent, together with consent from family members. The exclusion criteria were as follows: (1) comorbid depressive disorder, bipolar disorder, or other psychiatric disorders; (2) pregnancy or lactation; (3) severe physical illness; and (4) a history of psychoactive substance abuse. The exclusion of patients with a history of psychoactive substance abuse was prespecified to reduce potential confounding and clinical heterogeneity, because substance-related psychopathology and physiological effects may influence suicidal ideation, psychotic symptom severity, and peripheral biochemical or metabolic measures. Type 2 diabetes mellitus, renal dysfunction, and severe metabolic disorders were not specified as separate exclusion criteria. Although severe physical illness was an exclusion criterion, the available analytic dataset did not retain sufficiently detailed disease-specific screening information to verify whether these conditions had been systematically excluded.

This study was approved by the Ethics Committee of Shanghai Jing’an District Mental Health Center (Approval No. JAMHC-IRB-2021113). All participants and their family members provided written informed consent before enrollment. The study was conducted in accordance with the principles of the Declaration of Helsinki.

### 2.2 Measuring instruments

#### 2.2.1 General data

Demographic and clinical information was collected using a structured case-report form. The variables included sex, age, duration of illness, years of education, smoking duration, and constipation status. A separate measure of treatment duration (e.g., cumulative antipsychotic exposure) was unavailable in the analytic dataset. Although illness duration was recorded, it was not used as a proxy for treatment duration because it does not necessarily reflect cumulative or continuous treatment exposure. Data on body mass index, waist circumference, blood pressure, renal function indicators such as creatinine or estimated glomerular filtration rate, detailed antipsychotic type and dose, clozapine use, valproate use, benzodiazepine use, alcohol intake, purine-rich dietary intake, diuretic treatment, and urate-lowering treatment were not available in the analytic dataset. Verified diagnoses of type 2 diabetes mellitus, renal dysfunction, and severe metabolic disorders were also unavailable in the analytic dataset. These variables are recognized as major potential confounders of serum uric acid concentrations; therefore, the uric-acid analyses were designed and interpreted as limited-adjustment exploratory analyses rather than fully confounder-adjusted biomarker or mechanistic analyses. Clinical assessment and fasting blood sampling were intended to be completed at enrollment. However, the analytic dataset did not retain the exact dates or timestamps required to calculate the interval between PANSS/BSS/BSI-CV assessment and blood sampling; therefore, the mean or median interval and the proportion of participants with an interval > 2 days could not be quantified.

#### 2.2.2 Psychiatric symptoms and current suicidal ideation

Psychiatric symptoms were assessed using the PANSS. The PANSS total score and the positive, negative, and general psychopathology subscale scores were calculated, with higher scores indicating more severe psychotic symptoms.

Suicidal ideation was evaluated on the day of enrollment using the Chinese version of the BSS/BSI-CV. The Scale for Suicide Ideation was originally developed by Beck and colleagues (47) and later published as a copyrighted clinical assessment instrument (48). The Chinese version of the Scale for Suicide Ideation has been psychometrically evaluated in Chinese populations (49), and the BSS/BSI-CV has also been used in recent Chinese schizophrenia samples (10, 11, 13, 14). The present manuscript does not reproduce copyrighted item wording. The assessment referred to suicidal ideation during the preceding week, including the day of assessment. The first five items constitute a screening section for suicidal ideation. Within this screening section, item 4 assesses active suicidal desire or ideation, whereas item 5 assesses passive suicidal desire, such as a wish that death might occur through an external or accidental event.

Each BSS/BSI-CV item is scored from 0 to 2, with higher scores indicating greater suicidal ideation or suicide-related risk. For the primary analysis, the outcome was current suicidal ideation during the preceding week. Item 4 assesses active suicidal desire, whereas item 5 assesses passive suicidal desire. The prespecified outcome combined responses to these two items; therefore, passive-only and active suicidal ideation were not analyzed as separate subgroups. Patients who scored 0 on both items 4 and 5 were classified as having no suicidal ideation, whereas patients with any nonzero response on either item were classified as having suicidal ideation. This dichotomous definition was chosen to identify a clinically practical screening-defined outcome rather than to model suicidal-ideation severity.

All PANSS and BSS/BSI-CV assessments were conducted by experienced psychiatrists. Before the study assessments, raters received standardized training on scale administration, reviewed scoring anchors, and discussed scoring rules to improve procedural consistency. Clinical interviews were completed at enrollment, generally before laboratory results were available; however, formal documentation of rater blinding to biochemical data was not maintained. Laboratory personnel were separate from the clinical raters and were blinded to participants’ suicidal-ideation status.

### 2.3 Blood sampling and biochemical measurements

Morning fasting venous blood samples were collected after enrollment. Samples were centrifuged at 3,000 r/min for 15 min, and serum was separated and stored at − 80 °C until analysis. Blood-based biochemical, thyroid, lipid, and metabolic indicators were measured in the hospital clinical laboratory using an automated biochemical analyzer and chemiluminescence assay system (A2000Plus), according to standard operating procedures.

The measured indicators included triiodothyronine (T3), thyroxine (T4), free triiodothyronine (FT3), free thyroxine (FT4), thyroid-stimulating hormone (TSH), prolactin, erythrocyte sedimentation rate (ESR), serum uric acid, fasting glucose, triglycerides, total cholesterol, high-density lipoprotein cholesterol (HDL-C), low-density lipoprotein cholesterol (LDL-C), apolipoprotein A, apolipoprotein B, lipoprotein(a), and 25-hydroxyvitamin D [25(OH)D].

### 2.4 Quality control and variable definitions

To reduce measurement bias, clinical rating scales were administered by trained psychiatrists using standardized procedures. For the BSS/BSI-CV outcome, raters followed the predefined items 4/5 rule described above. Biochemical tests were performed in the same clinical laboratory, and laboratory personnel were blinded to participant suicidal-ideation status.

For descriptive and exploratory sensitivity analyses, selected continuous variables were categorized into clinically meaningful categories or sample-specific tertiles. Age, PANSS total score, serum uric acid, and thyroid function indicators were grouped into tertiles. Lipid categories were defined according to the National Cholesterol Education Program Adult Treatment Panel III thresholds, converted to millimoles per liter (mmol/L). Fasting glucose was categorized as normal (< 5.6 mmol/L) or elevated (≥ 5.6 mmol/L). The 25(OH)D status was classified as deficiency (< 12 ng/mL), inadequacy (12 to < 20 ng/mL), or adequacy (≥ 20 ng/mL). Hyperuricemia was defined according to the laboratory reference standards. Missing biochemical data were handled using available-case analysis. Missing data occurred for 25(OH)D, for which 106 of 134 patients had available measurements; the reason for missingness could not be fully reconstructed from the analytic file and is therefore acknowledged as a limitation.

### 2.5 Statistical analysis

Statistical analyses were performed using Python 3.11 with standard scientific-computing libraries, including scipy, statsmodels, scikit-learn, and matplotlib. Patients were divided into suicidal-ideation and nonsuicidal-ideation groups according to the prespecified definition of current suicidal ideation described above. Continuous variables were summarized as median (interquartile range) (M [P25, P75]) because most distributions were skewed, and between-group comparisons were performed using the Mann–Whitney *U* test. Categorical variables were presented as frequencies and percentages (*n* [%]) and compared using the chi-square test or Fisher’s exact test, as appropriate. Given the limited number of suicidal-ideation events and the available covariates, the primary multivariable logistic regression model was prespecified as a limited-adjustment model including PANSS total score, serum uric acid, age, and sex. PANSS total score and serum uric acid were scaled to 10-point and 100-µmol/L increases, respectively. No data-driven variable selection was used for the primary model. Treatment duration was not included in the multivariable analyses because this information was unavailable.

No additional confounder-adjustment or sensitivity model including the major determinants of serum uric acid described above could be fitted because these factors were unavailable. Accordingly, serum uric acid estimates were treated as limited-adjustment associations.

Other clinical and biochemical variables were analyzed descriptively and considered exploratory. ROC curve analysis was performed only as an auxiliary exploratory description of apparent discrimination for PANSS total score, serum uric acid, and the limited-adjustment, age- and sex-adjusted combined logistic model for current suicidal ideation. AUCs were reported with 95% confidence intervals estimated from 3,000 nonparametric bootstrap resamples. This resampling quantified uncertainty in the AUC estimates; it was not used to estimate or correct for optimism. Sample-derived Youden cutoffs were reported for descriptive purposes. The age- and sex-adjusted combined model was based on predicted probabilities from the logistic equation logit(*p*) = *β*0 + *β*1 × (PANSS total score/10) + *β*2 × (serum uric acid/100) + *β*3 × age + *β*4 × sex. All statistical tests were two-tailed, and *p* < 0.05 was considered statistically significant.

## 3 Results

### 3.1 Baseline characteristics and grouped biochemical profiles

A total of 134 patients with schizophrenia were included in the final analytic sample. Among them, 23 patients (17.2%) were classified as having suicidal ideation and 111 (82.8%) as having no suicidal ideation. Baseline demographic and clinical characteristics are presented in **Table 1**.

**Table 1:** Demographic and clinical characteristics according to suicidal-ideation status.

Characteristic	Overall (*n* = 134)	No suicidal ideation (*n* = 111)	Suicidal ideation (*n* = 23)	*p*-value
Age (years)	42.00 (36.00, 49.75)	42.00 (36.00, 48.00)	43.00 (37.50, 51.50)	0.490
Illness duration (years)	14.00 (9.00, 17.00)	14.00 (8.50, 17.00)	14.00 (11.50, 18.50)	0.325
Education (years)	9.00 (6.00, 12.00)	9.00 (6.00, 12.00)	9.00 (6.00, 12.00)	0.788
Smoking duration (years)	0.00 (0.00, 9.75)	0.00 (0.00, 10.00)	0.00 (0.00, 3.00)	0.535
PANSS total score	76.00 (69.00, 82.00)	75.00 (67.50, 81.00)	82.00 (77.50, 88.00)	< 0.001
PANSS positive score	26.00 (21.00, 29.75)	26.00 (20.00, 28.00)	27.00 (24.00, 33.00)	0.007
PANSS negative score	18.00 (15.00, 23.00)	18.00 (15.00, 22.00)	22.00 (14.00, 25.00)	0.192
PANSS general psychopathology score	26.00 (22.00, 31.00)	26.00 (22.00, 31.00)	30.00 (28.00, 33.00)	0.017
Sex				
Female	58 (43.3)	53 (47.7)	5 (21.7)	0.022
Male	76 (56.7)	58 (52.3)	18 (78.3)	
Constipation				
No	64 (47.8)	53 (47.7)	11 (47.8)	0.995
Yes	70 (52.2)	58 (52.3)	12 (52.2)	
Age tertile				
Low (≤ 38.00)	46 (34.3)	38 (34.2)	8 (34.8)	0.580
Middle (38.00–45.00)	46 (34.3)	40 (36.0)	6 (26.1)	
High (> 45.00)	42 (31.3)	33 (29.7)	9 (39.1)	
PANSS tertile				
Low (≤ 72.00)	46 (34.3)	42 (37.8)	4 (17.4)	< 0.001
Middle (72.00–80.67)	43 (32.1)	40 (36.0)	3 (13.0)	
High (> 80.67)	45 (33.6)	29 (26.1)	16 (69.6)	

Data are median (IQR) or *n* (%). *p*-values were derived using the Mann–Whitney *U* test, Pearson Chi-square test, or Fisher’s exact test, as appropriate.

PANSS, Positive and Negative Syndrome Scale.

Compared with the nonsuicidal-ideation group, the suicidal-ideation group had a higher proportion of male participants (78.3% vs. 52.3%; *p* = 0.022). Psychiatric symptom burden was also greater in the suicidal-ideation group, as reflected by higher PANSS total scores (82.00 [77.50, 88.00] vs. 75.00 [67.50, 81.00]; *p* < 0.001), PANSS positive scores (*p* = 0.007), and PANSS general psychopathology scores (*p* = 0.017). Age, illness duration, education years, smoking duration, constipation, and PANSS negative scores did not differ significantly between groups.

Biochemical and grouped metabolic profiles are shown in **Table 2**. Serum uric acid was significantly higher in the suicidal-ideation group than in the nonsuicidal-ideation group (349.00 [324.50, 373.00] vs. 302.00 [257.50, 351.50] µmol/L; *p* < 0.001), and the high uric-acid tertile was more frequent among patients with this outcome (56.5% vs. 28.8%; *p* = 0.008). Apolipoprotein A was lower in the suicidal-ideation group (0.88 [0.77, 0.99] vs. 0.96 [0.85, 1.13] g/L; *p* = 0.019). No statistically significant differences were observed for thyroid tertiles, lipid categories, dyslipidemia status, fasting glucose category, or 25(OH)D status (all *p* > 0.05).

**Table 2:** Biochemical, thyroid, lipid, and metabolic profiles according to suicidal ideation status.

Characteristic	Overall (*n* = 134)	No suicidal ideation (*n* = 111)	Suicidal ideation (*n* = 23)	*p*-value
T3	0.76 (0.64, 0.90)	0.76 (0.64, 0.91)	0.72 (0.64, 0.81)	0.392
T4	92.27 (74.79, 112.37)	92.19 (74.48, 113.83)	93.83 (82.56, 107.97)	0.859
FT3	3.68 (2.78, 4.35)	3.75 (2.83, 4.38)	3.56 (2.77, 4.26)	0.779
FT4	14.38 (12.68, 16.70)	14.23 (12.68, 16.77)	15.23 (12.89, 15.75)	0.839
TSH	2.75 (1.61, 4.12)	2.73 (1.65, 4.17)	2.91 (1.39, 3.81)	0.734
Prolactin	678.58 (456.51, 935.42)	698.76 (460.61, 945.42)	580.39 (369.02, 802.49)	0.110
ESR	22.00 (10.00, 45.00)	21.00 (9.50, 44.50)	23.00 (13.50, 47.00)	0.626
Uric acid (µmol/L)	313.50 (265.00, 356.00)	302.00 (257.50, 351.50)	349.00 (324.50, 373.00)	< 0.001
Glucose (mmol/L)	4.98 (4.43, 5.75)	4.99 (4.46, 5.77)	4.96 (4.34, 5.41)	0.608
Triglycerides (mmol/L)	1.62 (1.05, 2.52)	1.57 (1.02, 2.33)	1.94 (1.21, 2.95)	0.419
Total cholesterol (mmol/L)	4.63 (3.96, 5.34)	4.64 (3.99, 5.34)	4.44 (3.67, 5.33)	0.658
HDL-C (mmol/L)	1.18 (0.96, 1.43)	1.20 (0.96, 1.44)	1.09 (0.96, 1.39)	0.436
LDL-C (mmol/L)	2.66 (2.21, 3.50)	2.64 (2.24, 3.49)	2.87 (2.16, 3.45)	0.768
Apolipoprotein A (g/L)	0.96 (0.84, 1.12)	0.96 (0.85, 1.13)	0.88 (0.77, 0.99)	0.019
Apolipoprotein B (g/L)	0.89 (0.70, 1.08)	0.88 (0.70, 1.08)	0.92 (0.79, 1.02)	0.452
Lipoprotein(a) (mg/L)	167.50 (89.00, 254.00)	175.00 (94.00, 256.00)	147.00 (74.00, 246.50)	0.399
25(OH)D (ng/mL)	17.89 (12.97, 21.03)	18.02 (12.56, 20.81)	15.77 (13.88, 21.38)	0.920
Uric acid tertile				
Low (≤ 294.00)	46 (34.3)	44 (39.6)	2 (8.7)	0.008
Middle (294.00–340.67)	43 (32.1)	35 (31.5)	8 (34.8)	
High (> 340.67)	45 (33.6)	32 (28.8)	13 (56.5)	
Hyperuricemia				
No	118 (88.1)	97 (87.4)	21 (91.3)	1.000
Yes	16 (11.9)	14 (12.6)	2 (8.7)	
T3 tertile				
Low (≤ 0.68)	47 (35.1)	38 (34.2)	9 (39.1)	0.449
Middle (0.68–0.85)	43 (32.1)	34 (30.6)	9 (39.1)	
High (> 0.85)	44 (32.8)	39 (35.1)	5 (21.7)	
T4 tertile				
Low (≤ 81.49)	45 (33.6)	39 (35.1)	6 (26.1)	0.666
Middle (81.49–102.95)	44 (32.8)	35 (31.5)	9 (39.1)	
High (> 102.95)	45 (33.6)	37 (33.3)	8 (34.8)	
FT3 tertile				
Low (≤ 3.28)	45 (33.6)	38 (34.2)	7 (30.4)	0.779
Middle (3.28–4.10)	44 (32.8)	35 (31.5)	9 (39.1)	
High (> 4.10)	45 (33.6)	38 (34.2)	7 (30.4)	
FT4 tertile				
Low (≤ 13.44)	45 (33.6)	39 (35.1)	6 (26.1)	0.243
Middle (13.44–15.75)	44 (32.8)	33 (29.7)	11 (47.8)	
High (> 15.75)	45 (33.6)	39 (35.1)	6 (26.1)	
TSH tertile				
Low (≤ 1.92)	45 (33.6)	37 (33.3)	8 (34.8)	0.939
Middle (1.92–3.67)	44 (32.8)	36 (32.4)	8 (34.8)	
High (> 3.67)	45 (33.6)	38 (34.2)	7 (30.4)	
Triglyceride category				
Normal (< 1.70)	73 (54.5)	63 (56.8)	10 (43.5)	0.480
Borderline (1.70–2.25)	21 (15.7)	17 (15.3)	4 (17.4)	
High (≥ 2.26)	40 (29.9)	31 (27.9)	9 (39.1)	
Total cholesterol category				
Desirable (< 5.18)	91 (67.9)	75 (67.6)	16 (69.6)	0.756
Borderline (5.18–6.21)	35 (26.1)	30 (27.0)	5 (21.7)	
High (≥ 6.22)	8 (6.0)	6 (5.4)	2 (8.7)	
HDL-C category				
Low (< 1.03)	49 (36.6)	38 (34.2)	11 (47.8)	0.419
Intermediate (1.03–1.54)	66 (49.3)	56 (50.5)	10 (43.5)	
High/protective (≥ 1.55)	19 (14.2)	17 (15.3)	2 (8.7)	
LDL-C category				
Optimal (< 2.59)	65 (48.5)	54 (48.6)	11 (47.8)	0.939
Near optimal (2.59–3.36)	29 (21.6)	23 (20.7)	6 (26.1)	
Borderline high (3.37–4.13)	19 (14.2)	16 (14.4)	3 (13.0)	
High (≥ 4.14)	21 (15.7)	18 (16.2)	3 (13.0)	
Any dyslipidemia				
No	32 (23.9)	27 (24.3)	5 (21.7)	0.791
Yes	102 (76.1)	84 (75.7)	18 (78.3)	
Fasting glucose category				
Normal (< 5.6)	93 (69.4)	75 (67.6)	18 (78.3)	0.311
Elevated (≥ 5.6)	41 (30.6)	36 (32.4)	5 (21.7)	
25(OH)D status				
Deficiency (< 12)	24 (22.6)	20 (23.3)	4 (20.0)	0.922
Inadequacy (12 ≤ 20)	49 (46.2)	39 (45.3)	10 (50.0)	
Adequacy (≥ 20)	33 (31.1)	27 (31.4)	6 (30.0)	

Data are median (IQR) or *n* (%). Tertiles are sample-specific; lipid categories follow NCEP ATP III thresholds. Percentages for 25(OH)D are based on available data (*n* = 106).

FT3, free triiodothyronine; FT4, free thyroxine; TSH, thyroid-stimulating hormone; ESR, erythrocyte sedimentation rate; HDL-C, high-density lipoprotein cholesterol; LDL-C, low-density lipoprotein cholesterol.

### 3.2 Logistic regression analyses

Prespecified logistic regression analyses are summarized in **Table 3**. In crude models, PANSS total score and serum uric acid were significantly associated with current suicidal ideation. Each 10-point increase in PANSS total score was associated with higher odds of this outcome (OR = 2.23, 95% CI: 1.41–3.52; *p* < 0.001), and each 100-µmol/L increase in serum uric acid was similarly associated with higher odds of this outcome (OR = 2.41, 95% CI: 1.20–4.82; *p* = 0.013).

**Table 3:** Logistic regression models for current suicidal ideation, with emphasis on covariates in the limited-adjustment primary model.

Model	Variable	*N*	OR (95% CI)	*p*-value
Crude	PANSS total score, per 10-point increase	134	2.23 (1.41, 3.52)	< 0.001
Crude	Uric acid, per 100-µmol/L increase	134	2.41 (1.20, 4.82)	0.013
Limited-adjustment single-exposure model	PANSS total score, per 10-point increase	134	2.05 (1.29, 3.26)	0.002
Limited-adjustment single-exposure model	Uric acid, per 100-µmol/L increase	134	2.50 (1.20, 5.22)	0.015
Primary limited-adjustment combined model	PANSS total score, per 10-point increase	134	2.05 (1.27, 3.31)	0.003
Primary limited-adjustment combined model	Uric acid, per 100-µmol/L increase	134	2.46 (1.16, 5.25)	0.019
Primary limited-adjustment combined model	Age, per year	134	1.03 (0.98, 1.08)	0.322
Primary limited-adjustment combined model	Male sex vs. female	134	2.51 (0.82, 7.73)	0.108

The primary combined model included PANSS total score, serum uric acid, age, and sex. ORs are reported per 10-point increase in PANSS total score, per 100-µmol/L increase in uric acid, per year of age, and as male vs. female. Given 23 outcome events and the unavailability of uric-acid-related confounders, multivariable estimates are exploratory.

PANSS, Positive and Negative Syndrome Scale; OR, odds ratio; CI, confidence interval.

After limited adjustment for age and sex, both associations remained significant. In the primary combined model including PANSS total score, uric acid, age, and sex, PANSS total score (OR per 10-point increase = 2.05, 95% CI: 1.27–3.31; *p* = 0.003) and uric acid (OR per 100-µmol/L increase = 2.46, 95% CI: 1.16–5.25; *p* = 0.019) remained associated with current suicidal ideation. In the same primary model, age was not significantly associated with this outcome (OR per 1-year increase = 1.03, 95% CI: 0.98–1.08; *p* = 0.322), and male sex showed a nonsignificant positive association compared with female sex (OR = 2.51, 95% CI: 0.82–7.73; *p* = 0.108).

### 3.3 Auxiliary exploratory apparent ROC analysis

ROC results for the individual indicators and the age- and sex-adjusted combined logistic model are summarized in **Table 4** and **Figure 1**. The AUCs for PANSS total score and serum uric acid were 0.727 (95% CI: 0.601–0.839) and 0.722 (95% CI: 0.617–0.817), respectively. The combined model, which included PANSS total score, serum uric acid, age, and sex, yielded the highest AUC (0.768, 95% CI: 0.657–0.865). The corresponding AUC differences were 0.041 relative to PANSS total score and 0.046 relative to serum uric acid. At the sample-derived Youden cutoff (predicted probability, 0.116), the combined model yielded 91.3% sensitivity and 54.1% specificity, identical to the values for serum uric acid alone at its sample-derived cutoff.

**Table 4:** Auxiliary exploratory apparent ROC performance for discriminating between patients with and without current suicidal ideation.

Predictor	AUC (95% CI)	*p*-value	Sample-derived exploratory cutoff	Sensitivity (%)	Specificity (%)	Youden index
PANSS total score	0.727 (0.601, 0.839)	< 0.001	81.00	69.6	73.9	0.434
Uric acid	0.722 (0.617, 0.817)	< 0.001	312.00	91.3	54.1	0.454
Combined model	0.768 (0.657, 0.865)	< 0.001	0.116	91.3	54.1	0.454

The combined model included PANSS total score, serum uric acid, age, and sex. The 95% CIs for AUCs were estimated from 3,000 bootstrap resamples. Cutoffs were sample-derived using the Youden index; 0.116 denotes the predicted probability cutoff for the combined model.

ROC, receiver operating characteristic; AUC, area under the curve; CI, confidence interval.

**Figure 1:**
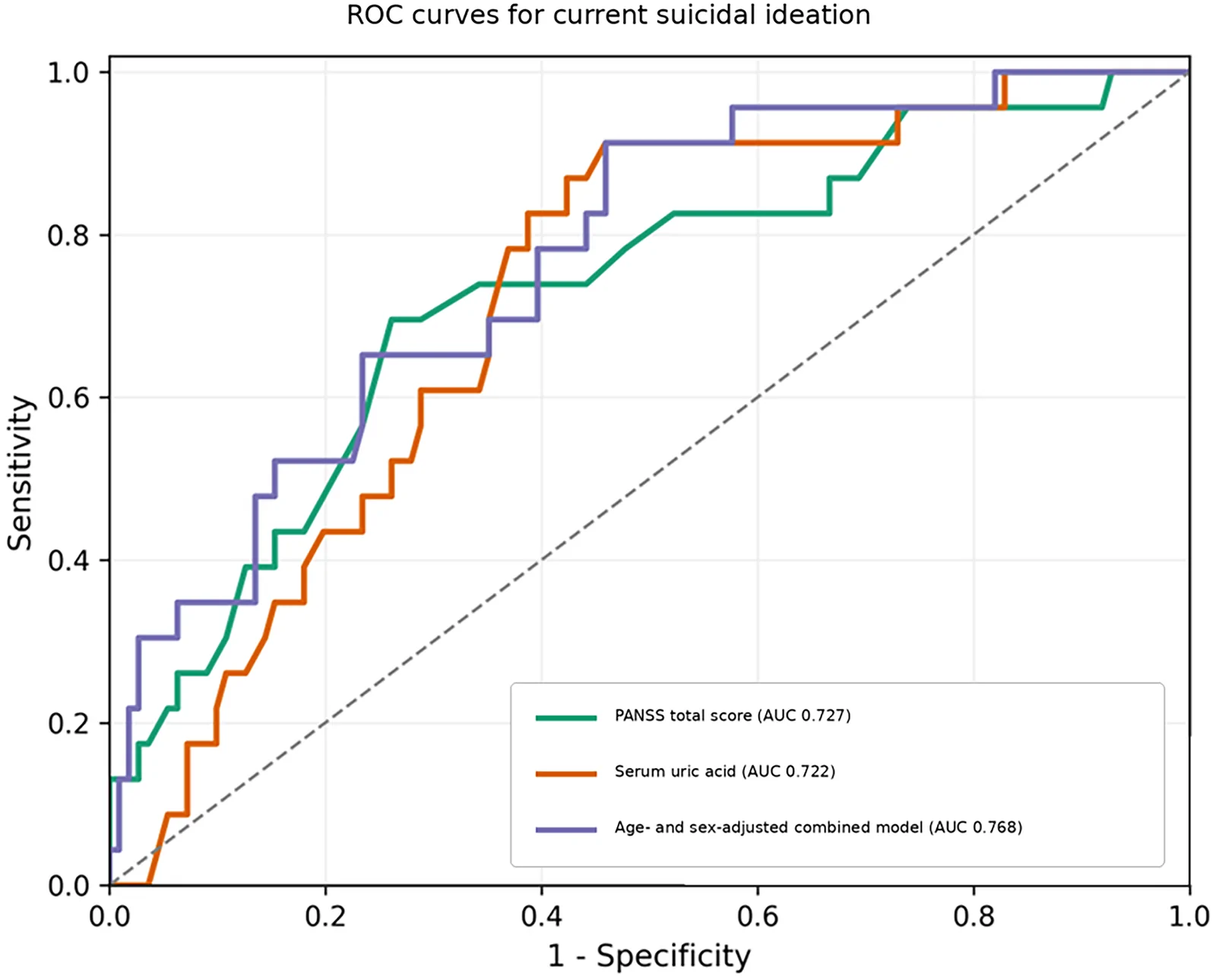
Auxiliary exploratory receiver operating characteristic curves showing apparent discriminative performance of PANSS total score, serum uric acid, and the age- and sex-adjusted combined logistic model including PANSS total score and serum uric acid for current suicidal ideation. The diagonal dashed line indicates no discrimination.

## 4 Discussion

In this cross-sectional study of 134 patients with schizophrenia, suicidal ideation was present in 17.2% of participants. Patients with suicidal ideation had greater overall psychopathology, including higher PANSS total, positive, and general psychopathology scores, as well as higher serum uric acid. In the prespecified model adjusted for age and sex, both PANSS total score and serum uric acid remained associated with current suicidal ideation. The combined model yielded the highest AUC, although the gain in apparent discrimination over either indicator alone was modest. These results suggest that clinical symptom burden and a routine biochemical measure may provide complementary descriptive information, but the findings remain exploratory.

The association between higher PANSS total score and suicidal ideation is compatible with the view that suicide-related vulnerability in schizophrenia is partly embedded in current psychopathology. In the present sample, positive and general psychopathology scores, but not negative symptom scores, differed significantly between groups. Recent studies in Chinese adults with schizophrenia have similarly linked suicidal ideation or tendencies to PANSS positive symptoms, general psychopathology, cognition, attention, insomnia, empathy, and aggression-related dimensions (10–17). Findings on negative urgency and emotion-related impulsivity further suggest that affective arousal and impaired emotion regulation may contribute to suicidal thoughts and behaviors in schizophrenia spectrum disorders (18, 19).

Serum uric acid was the most prominent biochemical correlate in the present analysis, but this finding warrants the greatest caution. Each 100-µmol/L increase in serum uric acid was associated with higher odds of suicidal ideation in both the age- and sex-adjusted single-exposure model and the primary combined model. However, serum uric acid concentrations are strongly influenced by sex, renal function, body mass index, waist circumference, blood pressure, diet, alcohol use, antipsychotic and mood-stabilizer exposure, diuretic or urate-lowering treatment, and broader metabolic status; several of these factors were unavailable in the analytic dataset. Therefore, the present study cannot determine whether the uric-acid association reflects suicide-related biological processes, renal or metabolic differences, medication-related effects, dietary factors, or residual confounding. Although uric acid is the end product of purine metabolism and an important circulating antioxidant and may be related to metabolic dysfunction, inflammatory activity, and symptom dimensions in schizophrenia (21–23), the current finding should be regarded as a limited-adjustment biochemical correlate rather than evidence of a mechanism. Oxidative-stress and inflammatory studies in schizophrenia and suicide-related psychiatric samples provide additional support for the broader clinical relevance of redox and immune-metabolic pathways (24–32), but they do not remove the need for direct confounder control in uric-acid analyses.

Several nonmutually exclusive mechanisms could plausibly link higher serum uric acid with suicidal ideation. Uric acid is the end product of purine metabolism and has a dual redox role. At physiological concentrations, it is an important extracellular antioxidant; however, increased purine degradation through xanthine oxidoreductase is accompanied by reactive oxygen species generation, and higher soluble urate may coexist with oxidative and inflammatory activation. In schizophrenia, oxidative stress and neuroinflammation have been associated with altered mitochondrial function, membrane integrity, and neurotransmitter regulation (21–32). One possibility is therefore that higher serum uric acid is not itself the direct cause of suicidal ideation but rather a peripheral marker of increased purine turnover and redox-inflammatory stress occurring alongside greater psychopathological burden. A second, nonexclusive possibility is compensatory: serum uric acid may increase in response to oxidative stress as part of an endogenous antioxidant response. These possibilities could also help explain why the direction of uric-acid associations differs across psychiatric disorders and clinical states. These mechanisms remain hypothetical because the present study did not measure xanthine oxidase activity, oxidative-stress or inflammatory mediators, or longitudinal uric-acid changes.

Clinically, this finding may be useful primarily as a prompt for integrated assessment rather than as a standalone biomarker. Serum uric acid is inexpensive and routinely measured. In a patient with schizophrenia who has marked current symptom burden, an unexpectedly high or increasing uric-acid concentration could prompt clinicians to ensure completion of a structured assessment of suicidal ideation and to review factors that can alter urate, including renal function, metabolic status, hydration, diet, and psychotropic medication exposure. Repeated uric-acid measurements interpreted alongside symptom trajectories may ultimately be more informative than a single measurement. However, the present data do not establish a clinical uric-acid cutoff, do not support urate-lowering treatment for suicide prevention, and should not replace direct suicide-risk assessment. Prospective studies are needed to determine whether serum uric acid adds clinically meaningful information beyond established symptom-based assessment.

Serum uric acid reference intervals are sex-, age-, and laboratory-dependent. In a study of healthy Chinese adults, the 2.5th–97.5th percentile reference intervals were 238–488 µmol/L for men and 155–364 µmol/L for women (50). Against this background, the 100-µmol/L unit used in our regression corresponds to approximately 1.68 mg/dL and represents a substantial shift within the usual adult distribution. In our sample, the overall median serum uric acid concentration was 313.50 µmol/L (IQR, 265.00–356.00), while the suicidal-ideation and nonsuicidal-ideation groups had median values of 349.00 µmol/L (IQR, 324.50–373.00) and 302.00 µmol/L (IQR, 257.50–351.50), respectively, a between-group median difference of 47 µmol/L. Thus, the odds ratio reported per 100-µmol/L increase should be understood as a statistical scaling choice that facilitates interpretation of the regression coefficient, not as a proposed clinical threshold or as evidence that every 100-µmol/L increase is pathologic. Whether an individual concentration is clinically abnormal depends on sex-specific laboratory reference limits and relevant renal, metabolic, dietary, and treatment factors.

Cross-diagnostic findings further caution against overinterpreting the direction of the present uric-acid association. Higher uric acid levels have been reported in bipolar disorder, particularly during manic states, whereas lower levels have been observed in major depressive disorder and in young patients with recent suicide attempts (43–45). Thus, the higher uric acid observed in our schizophrenia sample may reflect context-dependent interactions among redox state, inflammation, metabolic factors, and treatment exposure rather than a uniform psychiatric or suicide-related biological signal. Although experimental evidence implicates uric-acid-related redox and inflammatory signaling (46), these pathways were not measured in the present study and cannot be assumed to explain the observed association with suicidal ideation.

Apolipoprotein A was lower in the suicidal-ideation group in the univariate analysis. Because apolipoprotein A is closely related to HDL-mediated lipid transport and anti-inflammatory/antioxidant functions, whereas uric acid is linked to purine metabolism and redox homeostasis, the two markers may partly reflect overlapping metabolic processes and should not be interpreted as biologically independent. Given the exploratory nature of the apolipoprotein A finding, the limited number of suicidal-ideation events, and the absence of several relevant metabolic and renal confounders, we did not perform additional *post hoc* regression or ROC analyses or construct a uric-acid-to-apolipoprotein-A ratio. Larger studies are needed to determine whether apolipoprotein A provides information independent of serum uric acid.

No statistically significant differences were observed for thyroid-function tertiles, fasting glucose category, dyslipidemia status, or 25(OH)D status. These negative findings should be interpreted as absence of evidence in the present dataset rather than evidence that endocrine or metabolic systems are irrelevant to schizophrenia-related suicide risk. Thyroid hormone alterations in schizophrenia are heterogeneous and may vary by illness stage, medication exposure, and comorbid physical conditions (35). Vitamin D and broader metabolic profiles have also been discussed in relation to depression, anxiety, stress-related disorders, and psychiatric outcomes (36, 37). In the present study, the limited number of suicidal-ideation events, available-case analysis for 25(OH)D, and the use of broad categorical thresholds may have reduced the ability to detect subtle, nonlinear, or medication-dependent associations.

The ROC analysis suggested that combining PANSS total score and serum uric acid with age and sex provided only limited incremental discrimination over either indicator alone. At the sample-derived cutoff, the combined model did not improve sensitivity or specificity relative to serum uric acid alone. Accordingly, these ROC findings are best regarded as descriptive and hypothesis-generating (38, 39, 41).

This study has several strengths. First, the core outcome was explicitly operationalized as current suicidal ideation during the preceding week, providing a clinically practical binary screening outcome that captures both overt suicidal desire and passive death wishes. Second, the study integrated symptom severity with routine peripheral biochemical indicators, including thyroid, lipid, glucose, vitamin D, and uric-acid-related measures. Third, the primary model was prespecified and avoided data-driven selection despite multiple exploratory biomarkers, reducing the risk of overfitting. Finally, the analysis transparently reported the complete primary model covariates and explicitly acknowledged the low events-per-parameter ratio, which strengthens the interpretability of the findings.

Several limitations should be noted. First, the most important limitation for interpreting the uric-acid finding is unmeasured and residual confounding. Data on body mass index, waist circumference, blood pressure, renal function indicators such as creatinine or estimated glomerular filtration rate, detailed antipsychotic type and dose, clozapine exposure, valproate exposure, alcohol intake, purine-rich diet, diuretic treatment, urate-lowering treatment, and detailed metabolic syndrome components were unavailable. Illness duration was not used as a proxy for treatment duration because it may not reflect cumulative or continuous treatment exposure. Therefore, no additional confounder-adjusted model or sensitivity analysis including these factors could be performed. In addition, type 2 diabetes mellitus, renal dysfunction, and severe metabolic disorders were not available as verified diagnoses or disease-specific exclusions. Therefore, residual confounding by renal and metabolic conditions cannot be excluded, and the observed association between serum uric acid and suicidal ideation should not be interpreted as independent of these conditions. This substantially limits interpretation of serum uric acid and prevents strong biological, mechanistic, or independent-biomarker conclusions. Second, the primary outcome was a screening construct defined by items 4/5 of the BSS/BSI-CV rather than a suicidal-ideation severity measure. BSS total scores and severity-category data were not available, so description of total scores, severity modeling, or sensitivity analyses based on total scores could not be performed. Formal inter-rater reliability indices and formal blinding logs for clinical raters were also unavailable; therefore, measurement variability, outcome misclassification, or rater-related bias cannot be completely excluded. Third, the cross-sectional design precludes causal inference and does not determine whether higher uric acid preceded, accompanied, or followed suicidal ideation. Fourth, this was a single-center study with a modest sample size and only 23 suicidal-ideation events; therefore, the events-per-parameter ratio for the four-parameter primary model was low and multivariable estimates may be unstable. Fifth, the ROC analysis evaluated the combined model in the same sample in which it was developed. No optimism correction, calibration assessment, Brier score, decision-curve analysis, or external validation was performed; consequently, the AUC may be optimistic, and the model’s clinical utility remains unknown. The sample-derived cutoff therefore should not be used for clinical prediction or decision-making until the model has undergone adequate internal and external validation. Sixth, 25(OH)D data were incomplete (106 of 134 participants had available measurements), and the reason for missingness could not be fully reconstructed from the analytic file. Finally, multiple exploratory biochemical comparisons were performed without an adequately powered multiplicity-adjusted biomarker model; therefore, nonprimary findings, including apolipoprotein A, should be interpreted cautiously, and their independent or incremental value beyond the prespecified markers remains unknown.

An appropriate next step would be a sufficiently powered, multicenter prospective longitudinal cohort in which repeated BSS/BSI-CV and PANSS assessments are synchronized with fasting serum uric acid measurements and subsequent suicidal outcomes are prospectively recorded. Such studies should systematically collect the major determinants of serum uric acid that were unavailable in the present dataset, including renal function, body mass index, and other metabolic measures, detailed psychotropic exposure, alcohol and dietary factors, and diuretic or urate-lowering treatment, to determine whether the observed association persists after more complete confounder adjustment. Repeated paired measurements would also help clarify temporal ordering and whether within-person changes in serum uric acid track the emergence, persistence, or worsening of suicidal ideation. If the association is reproduced, subsequent studies should evaluate whether serum uric acid provides incremental value beyond established clinical variables using appropriately validated prediction models, followed by external validation in an independent schizophrenia cohort.

## 5 Conclusion

In this schizophrenia sample, current suicidal ideation during the preceding week was associated with greater PANSS symptom burden and higher serum uric acid after adjustment for age and sex. Serum uric acid should be interpreted as an exploratory biochemical correlate rather than a mechanistic risk factor. These findings support prospective evaluation of an integrated clinical–biochemical assessment approach but do not establish a model for clinical prediction.

## Data Availability

The raw data supporting the conclusions of this article will be made available by the authors, without undue reservation.
